# Impact of Peripheral Nerve Block Technique on Incidence of Phrenic Nerve Palsy in Shoulder Surgery

**DOI:** 10.1155/2023/9962595

**Published:** 2023-09-11

**Authors:** Aaron S. Campbell, Christopher D. Johnson, Shaun O'Connor

**Affiliations:** ^1^Centre for Biomedical Sciences Education, Queen's University, Belfast BT9 7AA, UK; ^2^Ulster Hospital, Dundonald BT16 1RH, UK

## Abstract

Peripheral nerve blocks are an increasingly common method of providing postoperative analgesia for shoulder surgeries. However, the standard technique, the interscalene block (ISB), inevitably causes hemidiaphragmatic paresis (HDP), secondary to phrenic nerve palsy. This can cause morbidity in patients with preexisting respiratory compromise, prompting investigation into alternative “phrenic-sparing” nerve blocks. The aim of this review was to give an overview of these blocks and critically evaluate the current literature to determine if any are suitable replacements for ISB. The incidence of HDP and analgesic efficacy were considered. We queried four electronic databases and one register. Twenty-eight original articles were selected for review. The use of ultrasound guidance, lower volumes of local anaesthetic (LA), and injection 4 mm outside the brachial plexus fascia reduced HDP incidence for the ISB; however, no single modification did so sufficiently. While the anterior suprascapular nerve block (SSNB) showed comparable analgesic effects to the ISB, HDP prevalence was also high. The posterior SSNB produced consistently low HDP incidences but also inferior analgesia to ISB, except when combined with an infraclavicular brachial plexus block. The superior trunk block (STB) provided equivalent analgesia to the ISB while reducing HDP incidence, but not significantly. Lower LA volumes consistently led to lower HDP incidence across all blocks, likely due to a reduced ability to spread to the phrenic nerve. Further investigation into the minimum effective volumes of the extrafascial ISB, anterior SSNB, STB, and combined posterior SSNB with infraclavicular block is warranted to determine if any of these blocks can successfully balance HDP prevention with analgesic efficacy.

## 1. Introduction

### 1.1. Background

Shoulder surgery encompasses a variety of procedures, both arthroscopic and open, including rotator cuff repairs, adhesiolysis, and total shoulder replacement [1]. Most procedures are now performed within the ambulatory setting, allowing for a more efficient and cost-effective process by reducing the number of in-patient procedures [2]. However, a challenging aspect of this system is postoperative pain, with an estimated 45% of patients experiencing severe pain following shoulder arthroscopy alone [3, 4]. It is essential that this pain is well controlled prior to discharge to allow for successful rehabilitation and prevent readmission [2]. Indeed, one study [5] attributed pain to 12% of readmissions following ambulatory procedures in nine different surgical specialties.

Regional anaesthesia has become an increasingly common method used to control this pain, either as the primary anaesthetic or as an adjunct to general anaesthesia (GA) [6]. Compared to GA alone, regional anaesthesia has shown to increase patient satisfaction while achieving similar pain scores [7, 8]. In addition, the introduction of these peripheral nerve blocks has reduced postoperative opioid consumption and, consequently, opioid-related side effects such as nausea and respiratory depression [9, 10].

The interscalene block (ISB) has become the “gold standard” regional anaesthesia technique for shoulder surgery [6, 11]. However, phrenic nerve (PN) palsy and subsequent hemidiaphragmatic paresis (HDP) are an inevitable side effect of this block, removing it as an option for those surgical patients with a history of respiratory compromise, including chronic obstructive pulmonary disease (COPD), bronchial asthma, and COVID-19 [6, 12]. Ironically, this is the patient population which would most benefit from the avoidance of opioid-induced respiratory depression that regional anaesthesia allows [11]. As such, there has been growing interest in alternative, “phrenic-sparing” nerve blocks which could provide comparable shoulder analgesia to ISB while reducing incidences of PN palsy [6].

### 1.2. Aims

The aim of this review is to determine if any “phrenic-sparing” technique can produce equivalent analgesia to ISB while sparing the PN. This would help to ascertain the optimal regional anaesthesia technique for patients with respiratory compromise.

### 1.3. Innervation of the Shoulder

To successfully manage intra- and postoperative pain, nerves supplying the periosteum, articular surfaces, synovium, capsule, ligaments, and skin of the glenohumeral joint (GHJ) must be blocked [13].

Skin in this area receives cutaneous innervation from the axillary nerve and suprascapular nerve (SSN), branches of the brachial plexus (BP), alongside the supraclavicular nerves of the cervical plexus (which are blocked separately without impacting the PN). Osteological components of the joint also receive innervation from the axillary nerve and SSN, in conjunction with the musculocutaneous, long thoracic, and lateral pectoral nerves [14].

Sensory innervation of the joint capsule and ligaments can vary amongst individuals, although the SSN and axillary nerve tend to supply the majority of these structures, with the former thought to provide up to 70% of innervation to this area [15]. More minor contributions to the anterior capsule are made by the lateral pectoral nerve and musculocutaneous nerve, although the latter may provide no innervation to the capsule in some individuals [16]. The lower subscapular nerve provides sensation to parts of the medial aspect of the capsule [17, 18]. As such, Borgeat [7] posits that it is essential to block the SSN and axillary nerve as a minimum for arthroscopic shoulder surgery as they provide the vast majority of sensation to the area.

### 1.4. The Phrenic Nerve

**Figure 1:**
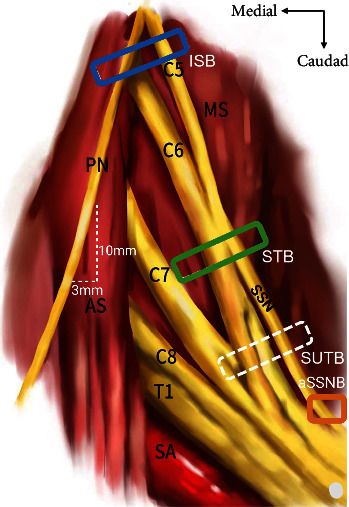
Schematic depicting the anatomical relationship of the left PN and BP, as well as the typical sites of injection for the ISB and phrenic-sparing blocks. PN, phrenic nerve; ISB, interscalene block site (blue rectangle); AS, anterior scalene; MS, middle scalene; SSN, suprascapular nerve; SA, subclavian artery; ST, superior trunk; STB, superior trunk block injection site (green rectangle); SUTB, subparaneural superior trunk block site (white rectangle); aSSNB, anterior suprascapular nerve block site (orange rectangle). Adapted from Zhang et al. [19].

The phrenic nerve (PN) is a peripheral nerve which contains fibres from the third to fifth cervical spinal nerves' anterior rami. Fibres of these three rami converge to form the nerve at the superolateral border of the scalenus anterior, where it then descends obliquely along the muscle's anterior surface, deep to the prevertebral fascia, as shown in Figure 1. The PN forms close to the roots of the BP, emerging between the anterior and middle scalene muscles, with only a thin fascia separating them [20, 21]. However, as the nerve travels caudally, the distance between it and the BP increases. Initially, the PN and BP are approximately 2 mm apart at the level of the cricoid cartilage (C6), before diverging by about 3 mm for every 1 cm that the PN descends within the neck [22]. The PN passes between the subclavian vessels in the root of the neck before entering the thorax via the thoracic inlet [11, 14]. Each PN then courses between the ipsilateral pleura and the fibrous pericardium, providing sensory innervation to both, along with the central diaphragm. They also provide the sole motor innervation to the diaphragm, with each PN innervating the ipsilateral hemidiaphragm independently [23].

### 1.5. Hemidiaphragmatic Paresis

As a result of being the only source of motor innervation to each hemidiaphragm, PN palsy can result in ipsilateral HDP [24]. The most relevant cause of this paresis is inadvertent PN blockade as a result of local anaesthetic (LA) spread from BP nerve blocks; the drug can spread anteriorly to reach the PN itself or proximally to block the C3–5 nerve roots [9].

In quiet inspiration, around 75% of tidal volume is a result of diaphragmatic movement [25]. Despite this, healthy adults with unilateral HDP are typically asymptomatic due to compensation by the contralateral hemidiaphragm [26, 27]. In contrast, those with comorbidities, most notably obesity and respiratory disease, can become dyspnoeic and require either noninvasive or invasive ventilation [28]. Phrenic nerve palsy can be diagnosed, and its severity measured, using several techniques. As mentioned, dyspnoea is the primary symptom of HDP, but it is neither sensitive nor specific for diagnosis [16].

Pulmonary function tests (PFTs) can be compared to preoperative baselines or predicted values to detect and quantify HDP [27]. This method measures forced expiratory volume in one second (FEV_1_) and forced vital capacity (FVC), each of which has been shown to decrease by over 25% as a result of unilateral PN palsy [29, 30]. However, PFTs give an indication of pulmonary function as a whole rather than the specific side that PN palsy is suspected [14].

Diaphragmatic ultrasound (US) has become widely used in detecting abnormal postoperative diaphragm movements due to its high sensitivity and specificity, coupled with the brief time it takes to perform and its noninvasive nature [31, 32]. Additionally, it can be used to quantify the degree of HDP and classify it as complete or partial by comparing it to a preoperative baseline diaphragmatic excursion [27].

In recent years, there have been various attempts to prevent HDP in those receiving regional anaesthesia of the shoulder, from modifications of the standard ISB to the development of more distal blocks, such as superior trunk block (STB) and suprascapular nerve block (SSNB).

### 1.6. Interscalene Block

#### 1.6.1. Landmark-Guided Lateral Approach

While attempts at BP blocks had been successful in the past, it was not until 1970 that the first consistently efficacious technique was created and has since become the standard technique [33]. Developed by Winnie [34], this lateral approach is performed at the level of C6, using the cricoid cartilage as a landmark. A finger is placed at the posterior border of the sternocleidomastoid at this level and “hooked” behind it to overlie the scalenus anterior. This finger is then drawn laterally along the muscle until the interscalene groove is reached. A needle can then be inserted into the groove at this level. Although the original technique involved needle insertion until paraesthesia was elicited, modern equipment such as nerve stimulators (NS) and US can now also be employed to ensure appropriate positioning between the C5 and C6 roots, within the BP sheath [16].

Conventionally, 15−20 mL of LA are injected [35]. Injection of LA at this point blocks the C5 and C6 nerve roots, whose fibres will go on to form the SSN and axillary nerve, the main sources of sensory innervation of the shoulder. As mentioned, LA spread can also lead to blockage of the PN, causing HDP [36]. Previously, HDP was seen as inevitability when using this block, with incidences of 100% [37, 38]. Since its inception, various modifications have been made to the Winnie [34] approach to reduce the incidence of this side effect. Modifications include a reduction in LA volume or concentration, the use of US or NS guidance, and changes to the needle approach itself.

A variation of this block was later developed by Meier et al. [39], with an injection site at the most proximal point of the interscalene groove and needle orientation towards the junction of the middle and lateral thirds of the clavicle.

#### 1.6.2. Ultrasound-Guided Lateral Approach

As anatomy is subject to variation, reliance on surface landmarks, such as in Winnie's [34] approach, can lead to complications such as block failure and damage to nearby structures. Consequently, this lateral approach is now typically performed with the aid of US guidance [40]. Using US, the cervical nerve roots can be seen as hypoechoic structures between the anterior and middle scalene muscles. A needle can then be passed through the middle scalene muscle to distribute LA around these nerve roots [41]. This technique is thought to have many advantages over landmark-guided ISB, with a study by Soeding et al. [42] showing improved block quality and reduced complications with the former.

#### 1.6.3. Extrafascial Technique

Albrecht et al. [43] investigated the maximum effective needle-to-nerve distance for ISB and concluded that needle contact with the nerve is not essential. In 85% of cases, the maximum effective distance for successful ISB was 5.2 mm lateral to the connective tissue sheath surrounding the BP. They concluded that it is possible to produce adequate analgesia while injecting outside of the BP connective tissue fascia/sheath.

Building on this, an “extrafascial” injection technique was developed. Whilst the initial needle approach is the same as any US-guided lateral approach, the final position of the needle tip is 4 mm lateral to the BP sheath, equidistant to the C5 and C6 nerve roots. This distance was chosen as it was calculated to be the maximum distance which would lead to a successful block in 90% of cases [30, 44].

### 1.7. Superior Trunk Block

The superior trunk of the BP is formed by the unification of the C5 and C6 nerve roots. As it travels distally, it gives rise to the SSN before dividing into anterior and posterior divisions [23]. First described by Burckett-St Laurent et al. [45], STB targets the C5 and C6 nerve roots at a more distal point than the ISB, with LA being injected at a point after these roots unite to form the superior trunk. Importantly, the trunk must be targeted prior to the exit of the SSN due to the large proportion of innervation that it provides to the shoulder. By targeting the BP at a more distal point than the ISB, LA injected will be further from the PN, reducing the chances of HDP [46]. Moreover, because all nerves innervating the shoulder emerge distal to this point, a similar analgesic effect to ISB should be achieved [47].

Like US-guided ISB, the C5 and C6 nerve roots can be identified in the interscalene groove using US. However, they are then tracked distally until the level at which they join to form the superior trunk, where a needle can then be inserted between the deep cervical fascia and scalenus medius muscle until it reaches the upper trunk [45]. The exact point of LA injection has been subject to variation, with the needle tip being placed lateral [45, 48], anterior, or posterior [49, 50] to the superior trunk.

Notably, this approach runs the risk of damaging the transverse cervical artery, which can lie directly superficial to the trunks of the BP [45]. The STB can also help to avoid mechanical injury to the dorsal scapular and long thoracic nerves which run through the middle scalene muscle. This is because, unlike in the ISB, the needle will travel superficially to the middle scalene rather than through it [47]. Although, in practice, it is not always possible to avoid passing through this muscle, encountering these nerves should still be considered a possibility [46].

A further advantage over the ISB, as highlighted by Burckett-St Laurent et al. [45], is that the superior trunk has a more consistent anatomical location than the C5 nerve root, which can take a variant course over or through the scalenus anterior in approximately 35% of individuals [51]. This allows for easier identification of the superior trunk on US [45].

More recently, a novel approach to the STB was attempted in a cadaveric study by Cros Campoy et al. [52], known as a subparaneural block. As the name suggests, this procedure involves the needle tip being placed within the paraneural sheath, between the anterior and posterior divisions of the superior trunk, as shown in Figure 1. An injection of 5 mL of dye at this level led to staining of the C5 and C6 nerve roots, the superior trunk and its divisions, as well as the SSN and lateral pectoral nerve. However, the PN remained unstained.

### 1.8. Suprascapular Nerve Block

First described by Wertheim and Rovenstine [53] for the alleviation of chronic shoulder pain, the SSNB has also been considered as a possible alternative to the ISB [54].

The SSN is a peripheral nerve which branches from the superior trunk of the BP approximately 3 cm superior to the clavicle, receiving fibres from C5, C6, and often C4 [23]. It then passes inferiorly via the posterior cervical triangle before travelling beneath the inferior belly of the omohyoid muscle [55]. From here, the SSN passes posterolaterally into the supraspinous fossa of the scapula via an opening between the suprascapular notch and the superior transverse scapular ligament [56]. Its terminal branches then pass through the spinoglenoid notch. It provides motor innervation to the supraspinatus and infraspinatus muscles and sensory innervation to about 70% of the GHJ capsule, as well as the acromioclavicular joint and subacromial bursa [57].

Due to the significant proportion of sensory innervation this nerve provides to the GHJ, two different variations of the SSNB have been developed: the anterior and posterior approaches.

#### 1.8.1. Anterior Approach

The anterior SSNB targets the BP more distally than either ISB or STB, so it is further still from the PN. It involves using an US probe to locate the SSN as it branches from the superior trunk. The SSN is then tracked until it passes under the inferior belly of the omohyoid. It is at this point that the needle is inserted and LA is injected [57, 58].

The inferior belly of the omohyoid is an easily identified landmark, allowing the SSN to be located more reliably than with the posterior approach. Furthermore, the more proximal injection site should ensure that all sensory branches of the SSN are sufficiently blocked. However, these benefits come with the potential risk of LA spreading to the BP and PN, which are much closer in proximity than in the posterior approach [59].

#### 1.8.2. Posterior Approach

The posterior approach to SSNB can be accomplished using anatomical landmarks [60], but it is now typically performed under US guidance in order to reduce the risk of complications like pneumothorax [61].

In this approach, the US probe is placed parallel to the scapular spine and passed superiorly to overlie the supraspinous fossa. From here, its position is adjusted until it reaches the suprascapular notch, allowing visualisation of the SSN passing deep to the superior transverse ligament. A needle can then be advanced in a lateral-to-medial direction and LA is injected to surround the nerve in the suprascapular fossa [57, 61].

As LA is injected at a much greater distance from the PN than in ISB, STB, or anterior SSNB, it should theoretically produce less PN palsy [62]. However, targeting so distally along the SSN's length has its own drawbacks as shown by a cadaveric study [63] which reported previously undescribed anatomy of the distal SSN. It was noted that one sensory branch of this nerve, the medial subacromial branch, arose proximal to the suprascapular notch in 57% of specimens. In addition, the lateral subacromial branch arose proximal to the notch in 43% of specimens. While only 14 cadavers were analysed and, like any cadaveric study, dissection may have altered the typical anatomy of this area, the presence of any sensory branches proximal to the site of injection implies this approach may fail to fully block these branches, causing inadequate analgesia.

In addition to this, the nerve can be difficult to visualise at this point using US as it is located deep to the supraspinatus muscle, increasing the likelihood of block failure [64].

#### 1.8.3. Combination Blocks

Whilst the SSN supplies the majority of the shoulder joint's sensation, the axillary nerve also provides an estimated 10% of shoulder sensation [15]. As such, this nerve can be blocked alongside the anterior or posterior SSNB to improve analgesic efficacy [65].

Arising from the posterior cord of the BP, the axillary nerve runs inferolaterally to the lateral border of the subscapularis, before entering the quadrangular space alongside the posterior circumflex humeral artery [66]. It can be blocked as it exits the quadrangular space using either a landmark or US guidance [65].

The SSNB can also be combined with blocks designed for more distal upper limb surgery, such as the supraclavicular and infraclavicular nerve blocks [67, 68].

The infraclavicular block targets the BP at the level of the cords [69], thus blocking their branches, including the axillary, lateral pectoral, and subscapular nerves, but sparing the SSN [70]. Therefore, it blocks the 30% of shoulder innervation not provided by the SSN, making it inappropriate in isolation for shoulder surgery but a theoretically useful adjunct to the SSNB.

The supraclavicular block targets the BP at the distal parts of its three trunks, with the aim of surrounding the plexus with LA [69].

## 2. Materials and Methods

### 2.1. Search Methods for Identification of Studies

A systematic search was conducted using five different electronic databases: Ovid Medline, Ovid Embase, Scopus, Web of Science, and the Cochrane Library. Specific search strategies were implemented for each electronic database. These strategies are documented in Appendix A. No limitations were placed on the language or publication period. The most recent searches were performed on 17 June, 2022. Due to the limited timeframe that the results of these search engines include, the reference lists of selected articles were also screened for additional studies. Search results were further supplemented with informal searches for key topic names within this field.

### 2.2. Study Inclusion and Exclusion Criteria

#### 2.2.1. Inclusion Criteria

Adults (aged 18 or over) undergoing any type of shoulder surgery receiving single-shot STB, SSNB, or modified ISB function had a measured outcome via symptoms, radiograph, US, and/or PFTs.

#### 2.2.2. Exclusion Criteria

Adolescents (aged under 18); subjects receiving continuous nerve blocks; studies analysing the efficacy of these nerve blocks for chronic pain management; case reports and case series; and ongoing trials.

### 2.3. Types of Outcome Measures

#### 2.3.1. Primary Outcomes

1. Overall incidence of HDP based on sonographic assessment
2. Decrease in hemidiaphragmatic excursion compared to baseline based on US assessment 30 minutes postblock
3. Percentage reduction from baseline in FVC 30 minutes postblock

#### 2.3.2. Secondary Outcomes

1. Volume and type of LA used
2. Postoperative pain scores
3. Total postoperative opioid consumption 24 hours postblock

### 2.4. Data Collection and Analysis

The titles and abstracts of the results yielded by these search strategies were screened after duplicate search results were removed. Irrelevant results were removed, and the full text of the remaining articles was retrieved for further screening. Reasons for exclusion at this stage were documented.

## 3. Results

### 3.1. Search Results

**Figure 2:**
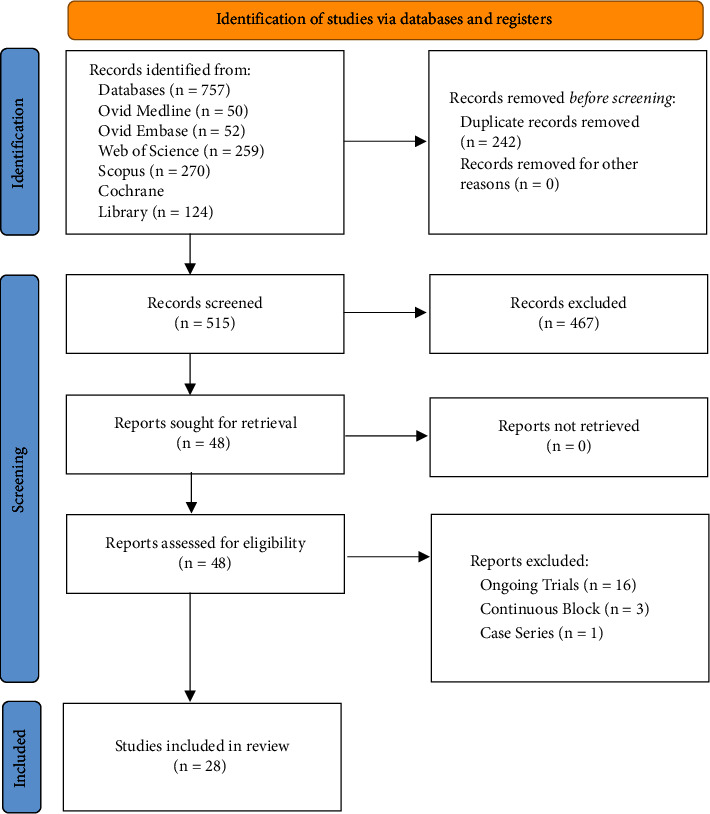
PRISMA flowchart summarising search strategy (*n* = number of studies) [71].

Our initial search of online databases and registers yielded a total of 757 studies, of which there were 515 original papers following the removal of duplicate search results. The titles and abstracts of these remaining articles were subsequently screened, and 467 were removed following the application of the inclusion and exclusion criteria. Full texts were retrieved for the remaining 48 articles. A total of 28 articles were included in the review. A summary of the search strategy is illustrated in Figure 2. Summaries of the characteristics of these studies are available in Appendix B.

### 3.2. Interscalene Block Results

Our search yielded 13 studies which directly compared the incidence of HDP in modified versions of the ISB. Summaries of their results are presented in Table 1. Thirteen additional studies were found in which standard ISB was used as a control to compare with SSNB or STB. These are discussed in the respective block's section.

Five studies compared ISBs of different volumes. Urmey and Gloeggler [72] reported incidences of 100% in both 20 mL and 45 mL landmark-guided blocks using 1.5% mepivacaine. However, two studies reported significant reductions in HDP incidence when 5 mL was injected under US guidance compared to a control ISB using 10 mL [74] and 20 mL of ropivacaine [73]. Trials by Sinha et al. [75] and Meena et al. [80] demonstrated similar HDP incidences for a 10 mL and 20 mL ISB performed under US and NS guidance, respectively. All of these groups reported statistically similar analgesia in the high- and low-volume study groups in the postoperative period.

Two studies modified both volume and concentration when studying ISB [78, 79]. Both studies used the same dose of LA for each block (50 mg) by adjusting the volume and concentration accordingly. Both demonstrated consistently high HDP incidences, regardless of the volume and concentration used, with incidences ranging from 58% to 70% across the five volume/concentration combinations used. In addition, in both studies, there was no statistically significant difference in postoperative pain scores, or rescue analgesia use amongst the study groups.

Palhais et al. [30] and Ayyanagouda [44] conducted studies comparing the injection of 20 mL of ropivacaine via the lateral approach to injecting the same volume using the extrafascial approach. In both cases, the extrafascial technique significantly reduced the incidence of HDP, with the added benefit of producing similar pain scores and opioid consumption postoperatively.

One study by Renes et al. [41] compared HDP incidence produced by an US-guided ISB to that produced by a NS-guided ISB. Notably, a Meier approach was utilised for the NS-guided blocks, whilst a lateral approach was utilised for the US group. The study showed significantly lower incidences of PN palsy in the US-guided group (13.3% vs 93.3%). Postoperative pain scores showed no statistically significant differences.

### 3.3. Superior Trunk Block Results

All STB studies yielded by our search are summarised in Table 2 on page 10. The earliest randomised control trial (RCT) with HDP as an outcome [47] showed that, despite STB causing HDP in significantly less patients than ISB, it still had overall HDP incidence rates of 76.3%. A later study by Kim et al. [48], using the same lateral injection site and LA volume produced similarly high results. In addition, different volumes of LA were compared in the latter study, with 5 mL of LA producing much lower rates of HDP (14.3%) when injected lateral to the superior trunk.

A larger trial using a 2-point posterior/anterior injection technique demonstrated HDP rates of just 4.8% for STB with 15 mL of LA, significantly less than the ISB [49]. However, a study using a similar injection technique [50] showed no significant difference between HDP incidence in ISB and STB groups. A subsequent study injecting 12 mL of bupivacaine deep (posterior) into the superior trunk also produced high incidences of HDP [81].

A recent study by Zhang et al. [19] has been the only one to investigate the subparaneural approach's efficacy in clinical practice, showing HDP incidences of 16.7% when using 5 mL of LA.

All but one RCT comparing STB to ISB found that STB provided noninferior analgesia and similar 24-hour opioid consumption levels. Only the study by Lee et al. [50] analysed anaesthetic effect of STB rather than purely its postoperative analgesic effect (i.e., its ability to be used without the use of GA) and found that STB produced a lower anaesthesia grade than ISB.

### 3.4. Suprascapular Nerve Block Results

Our search yielded five studies of the posterior SSNB with HDP incidence as an outcome. A summary of the data from all SSNB studies can be found in Table 3 on page 12. Only one article studied posterior SSNB without any additional blocks combined with it. This study demonstrated preservation of PFTs from baseline in those receiving either type of SSNB compared to those receiving ISB [85]. Likewise, when combined with a supraclavicular block [68] or infraclavicular block [67], respiratory function was maintained by the posterior approach, with incidences of HDP reaching 0%. However, one study did report a single instance of a posterior approach causing HDP. This was the only study which investigated a posterior SSNB combined with an axillary nerve block (AXNB) [62]. Despite this, overall HDP incidence was just 2% in this study.

Posterior SSNB displayed inferior analgesia to anterior SSNB when not coupled with any other blocks [85]. In the study which combined posterior SSNB with an infraclavicular nerve block [67], the ISB provided improved analgesia in the first 30 minutes postoperative but equivalent analgesia thereafter. However, combining the posterior approach with a supraclavicular block led to a comparable analgesic effect to ISB being achieved [68].

Four studies analysed the lone anterior SSNB's effect on the hemidiaphragm amongst other outcome measures. All studies which compared anterior SSNB to ISB consistently showed that the former caused significantly fewer cases of HDP in comparison to the latter, with incidences as low as 5.6% [82, 84, 86]. All four studies of the lone anterior SSNB showed that it provided noninferior analgesia and opioid consumption to the ISB.

Only two studies to date have measured HDP incidence as an outcome when studying the combined SSNB and AXNB. An RCT comparing this combination performed with either an anterior or posterior approach to SSNB was conducted by Ferré et al. [62]. In this study, 40% of patients receiving an anterior approach experienced some degree of HDP, compared to just 2% in the posterior group. However, the latter group experienced higher rates of opioid consumption postop. In a study by Rhyner et al. [87], an anterior SSNB combined with an AXNB was found to reduce the incidence of HDP when compared to the traditional ISB. Notably, the only study combining the anterior approach with an infraclavicular block [83] showed comparable postoperative analgesia use to ISB without any significant changes in spirometry readings postblock.

## 4. Discussion

### 4.1. Interscalene Nerve Block

The use of 10 mL or greater consistently produced high PN palsy rates. However, studies which used 5 mL of LA noted a significant reduction in HDP incidences [73, 74]. This can be attributed to the reduced ability of low volumes of LA to spread within the compartment into which they are injected. Such volumes may be unable to physically reach the PN and block it [35]. Whilst such low-volume injections are not currently standard practice due to concerns over inadequate analgesic efficacy [35], these studies demonstrated that they provide sufficient analgesia. The introduction of US-guided blocks, which have since become the standard of care [80], has allowed accurate deposition of LA directly adjacent to the target nerves, thus reducing the volume required. Recent studies have shown that, under US guidance, effective analgesia can be achieved with ISB using as little as 0.9 mL [88]. This smaller volume could theoretically reduce HDP incidence to an acceptable level; however, this is less likely when put into actual practice.

Renes et al. [41] also demonstrated that, even when depositing the same volumes of LA, US guidance significantly reduces the incidence of HDP compared to NS-guided blocks. This is due to the US allowing real-time visualisation of LA spread, so minor adjustments to needle position can be made during injection to ensure adequate coverage of the target nerve whilst minimising spread in the direction of nearby structures, including the PN [44]. However, both NS and US are preferable to landmark techniques [72]. The landmark technique consistently produced HDP in all study subjects, likely due to the larger volumes of LA required to ensure adequate nerve blockade in this “blind” technique.

The extrafascial injection technique's improved phrenic-sparing capabilities [30, 44] make sense anatomically as, in the intrafascial technique; the LA is injected into a more confined space, promoting proximal spread to the PN. Similar analgesia and block success rate to the classical approach was achieved, attributed to the ability to view the LA spread on US and adjust the needle as needed to ensure coverage of the C5 and C6 nerve roots (ibid).

In summary, no single modification to the “gold standard” lateral approach of ISB has managed to fully eliminate PN involvement. Whilst an US-guided, 10 mL lateral approach [41] has managed to reduce the incidence of HDP to as low as 13.3%, this prevalence is still too high to be used in clinical practice for those with respiratory compromise. Indeed, Tran et al. [11] liken prevention of PN palsy in these patients with prevention of pregnancy in those using contraception; it is an “all-or-nothing phenomenon,” so a phrenic-sparing option must produce incidences as close to 0% as possible. Further combining these different strategies may help lower HDP incidences closer to this target. For instance, the use of an extrafascial approach with low volume could, in theory, cause even less HDP than the current 20 mL extrafascial injection. However, further study into these combined modifications is required.

### 4.2. Superior Trunk Block

A wide range of HDP incidences is evident among STB studies, with incidences as low as 4.8% [49] and as high as 76.3% [47]. This discrepancy may be explained by the differing injection sites. However, these studies tended to produce HDP incidences closer to the higher end of this range. While these incidences were significantly lower than the ISB, as was to be expected from the greater distance between the injection site and PN, they are still too high for the STB to be useful in clinical practice for patients with impaired respiratory function. A possible anatomical explanation for this is the fact that, on average, the C5 and C6 nerve roots are only 43 mm and 50 mm in length, respectively, before they merge [89]. As such, the distance between the injection sites of the ISB and STB are of a similar length. As the PN only diverges 3 mm from the BP for every 1 cm it descends, the 50 mm more distal STB injection site would be only 1.5 mm further from the PN than the ISB injection site. Therefore, LA can still easily spread to the PN when injected at the superior trunk. The additional 1.5 mm it must spread from the ISB injection site could explain the increased rates of HDP with this block compared to STB.

Except for the subparaneural block, different injection sites appear to have little impact on HDP incidence, with needle positions posterior and lateral to the superior trunk producing similarly high results. However, there was one aberrant result, with a two-point posteroinferior/anterior injection technique used by Kim et al. [49] producing a HDP incidence of just 4.8%. These findings are contradicted by Lee et al. [50], whose study with a similar injection technique produced an incidence of 70.8%. The lack of reduction in HDP in this study may be partially attributed to the LA volume used, as they injected 10 mL anterior to the trunk rather than 5 mL, possibly increasing the risk of LA spread to the PN which lies more anterior than the BP [23]. However, it is unlikely that this fully explains such a significant difference in results, especially considering Robles et al. [81] produced HDP incidences of 60% using a solely posterior injection site (i.e., removing anterior injection completely) with only a marginally larger volume injected posteriorly compared to Kim et al. [49] (12 mL vs 10 mL).

Another possible explanation for the reduction in HDP incidence seen with the posteroinferior injection technique by Kim et al. [49] over the solely posterior injection sites in the other two studies is the existence of a fascia separating this point from the PN.

Rather than a continuous sheath surrounding the BP, it is believed that these nerves lie within a tissue plane, much like the sciatic nerve, with any connective tissue taking the form of convoluted septa, which can restrict the movement of injected dye or LA [90, 91]. These septa, along with variations in the muscle density of the scalene may impact the spread of LA in this region [43]. Whilst multiple cadaver studies have studied the connective tissue surrounding the BP [90, 92], the exact arrangement of these septa and the compartments they create has not been defined, only that they can be traced from as proximally as the scalenus anterior and medius and as distally as the upper humerus [93]. As the superior trunk exists within this area, it is possible that these septa are influencing LA movement during STB as well as other BP blocks. Again, due to the miniscule differences in these injection sites, it is unlikely this can fully explain the large discrepancies between the posterior injection sites' results.

In contrast to this, different LA volumes appeared to have a clear impact on HDP incidence, although only one paper studied this factor [48]. This is in keeping with similar studies of the impact of LA volume in ISB [73, 74].

The only study of the subparaneural approach's efficacy in clinical practice [19] corroborated the findings of a prior cadaveric study of this technique [52]. A possible explanation for this is that the paraneural sheath limits the spread of LA, preventing it from reaching the PN and forcing it to spread within the sheath cranially to the C5/6 nerve roots and SSN and caudally to the lateral pectoral nerve. This also allows for much smaller effective LA volumes [19]. However, it should be noted that this technique has the potential to increase the risk of mechanical nerve injury, although this did not occur in this study group. This risk likely makes the technique inappropriate for regular clinical practice.

All but one study in Table 3 demonstrated consistently noninferior analgesic effects and opioid consumption levels produced by STB compared to ISB; this implies that STB is an effective alternative to ISB. While some authors have noted the risk of reduced analgesic effect with a lower volume ISB [11], Kim et al. [48] demonstrated no significant differences in pain scores and rescue analgesia use when comparing a 5 mL STB to a 15 mL STB, despite the 5 mL injection having a higher median worst postoperative pain score. As such, we can hypothesise that use of 5 mL of LA is sufficient. However, a specific investigation into the minimum effective LA volume for this block is necessary to further reduce HDP incidence whilst maintaining adequate analgesia.

It should be noted that all published clinical trials examining STB have only used patient populations undergoing arthroscopic procedures. As established, ISB has been used as effective postoperative analgesia for open procedures as well as arthroscopic [30]. Again, the fact that STB should, in theory, block the same peripheral nerves as ISB leads us to hypothesise that it could also provide sufficient postoperative analgesia for more painful open procedures, unlike the more selective SSNB. An RCT comparing postoperative pain scores for ISB and STB in a patient population undergoing open procedures could test this hypothesis.

As the literature currently stands, STB produces lower incidences of HDP compared to ISB while maintaining analgesic efficacy. However, incidences remain too high for this block to be used as a phrenic-sparing block in clinical practice, although the use of a low-volume STB appears to have the potential to produce more clinically significant results [48].

A clinical trial directly comparing the various needle placements (posterior, anterior, and lateral) within one study population would help to confirm the ideal technique for STB and, indeed, whether needle position relative to the superior trunk actually has any meaningful impact on the PN. From there, determining the minimum effective LA volume using this ideal needle position would establish the full phrenic-sparing capabilities of this block.

### 4.3. Suprascapular Nerve Block

#### 4.3.1. Posterior Approach

From an anatomical perspective, the injection site in this approach (the suprascapular notch) is far enough from the path of the PN that the small volumes of LA used (the largest being 15 mL) cannot spread across the distance needed to affect this nerve [85], causing the PN sparing seen in these studies. However, as the SSN is blocked at a more distal point than in the ISB or anterior SSNB, it is possible that some of its more proximal sensory branches (e.g., the medial and lateral subacromial branches) remain unblocked and therefore are still able to convey pain sensation, reducing analgesic efficacy. As such, Taha [94] states that a combined posterior SSNB and AXNB provide sufficient analgesia for only minor operations.

In addition, Ferré et al. [62] reported one instance of a posterior approach causing HDP. They mention that, with this particular patient, they were unable to identify the SSN on US, which was likely due to its depth under the supraspinatus muscle, as mentioned earlier. As such, LA may have been injected more anteriorly to the suprascapular notch than necessary, close enough to the PN to allow LA to spread to it.

The study of the combined posterior SSNB and supraclavicular BP block [68] only utilised symptoms to diagnose HDP in their study group. As their study group was composed entirely of patients who were American Society of Anesthesiologists (ASA) status I or II, it is a possibility that this detection method underestimated HDP prevalence as healthier patients such as this are often asymptomatic with unilateral HDP. The true HDP incidence is likely higher than the 0% reported, especially considering lone supraclavicular blocks have been shown to produce HDP incidences of 44% [95].

The infraclavicular block has previously been shown to produce low HDP incidences (ibid). This was supported by the studies which combined it with a posterior [67] and anterior approach [83]. Notably, the latter reported noninferior analgesia compared to ISB and equivalent surgical anaesthesia (the only SSNB study to test this), while the former reported inferior analgesia compared to ISB but only for the first 30 minutes postoperative, after which they were comparable. This makes sense anatomically as the SSNB blocks the SSN while the infraclavicular block covers the other peripheral nerves innervating the shoulder (axillary, lateral pectoral, and subscapular) via blockage of the posterior and lateral cords of the BP from which they emerge.

#### 4.3.2. Anterior Approach

The anterior approach has consistently shown comparable analgesic effects to the ISB, especially when combined with an AXNB, whilst also causing significantly fewer incidences of HDP. This fact is corroborated by a number of systematic reviews [12, 96, 97]. Again, the more distal injection site to the ISB means the injectate has further to spread to block the PN. However, HDP incidence still reached 40% in one study [62]. Due to the severe risk that HDP can pose in those with compromised respiratory function, incidences of this size are too high for routine practice.

The reason for the higher HDP incidence reported by Ferré et al. [62] compared to other studies of the anterior approach are unclear. It is unlikely that the addition of an AXNB influenced the result as this nerve is far enough from the PN that LA spread is unlikely to cause HDP. A possible explanation was later provided by Lim et al. [85], who emphasised that, in their study, they followed the SSN as distally as possible on US and used this point as their injection site, maximising the distance of the LA from the PN whilst still injecting prior to the take-off of the SSN's branches. This resulted in the anterior and posterior approaches having comparable HDP incidences in their study. It is possible that Ferré et al. [62] targeted the nerve more proximally in its course and, thus, closer to the PN.

A recent cadaveric study [98] found that the minimum effective LA volume for complete circumferential coverage of the SSN whilst sparing the PN was just 4.2 mL. This is significantly lower than any volume used in studies of the anterior SSNB and has yet to be tested clinically. Therefore, the anterior approach may have the potential to provide equivalent analgesia to ISB (especially if combined with an AXNB) whilst preserving patient respiratory function to the same degree as posterior SSNB. An RCT testing the efficacy of a low-volume anterior SSNB targeted as distally along the SSN's course as possible compared to a standard anterior SSNB would help to determine whether it can be safely recommended for routine use in vulnerable patients.

### 4.4. Limitations

We utilised a systematic approach to our literature search in order to ensure we captured as many studies on this topic as possible, but without a full systematic review and meta-analysis, due to the broadness of the questions we were asking, with multiple block variations being studied and outcomes being assessed through a wide range of modalities (e.g., PN palsy being measured via complete hemidiaphragmatic paralysis, percentage reduction in hemidiaphragm excursion, changes in PFTs, or the presence of symptoms). In addition, studies varied in what blocks were compared, with some comparing the ISB to the SSNB, others comparing the ISB to the STB, and others still comparing different approaches to the same block. As such, it would have been difficult to collate enough homogenous data across the limited number of studies available to perform any meaningful comparison between the blocks as part of a meta-analysis. Although the costoclavicular block has emerged in recent years as a possible phrenic-sparing nerve block, we chose not to include it in our review due to the lack of studies focusing on its phrenic-sparing ability, with most instead focusing solely on its analgesic efficacy at the time of writing. As more data is accumulated on this block, however, it is likely that a further review incorporating it will be necessary.

## 5. Conclusions

While modifications to the “gold standard” ISB, such as lower LA volumes and extrafascial injection can help to reduce the incidence of PN palsy, none can reliably eliminate the incidence of this side effect entirely.

Similarly, alternative nerve blocks have shown a reduction of HDP incidence, albeit to varying degrees. On one end of the spectrum, posterior SSNB has consistently spared the PN, but at the cost of adequate analgesia for all but the most minor shoulder operations. At the other end of the spectrum, STB appears to produce similar analgesia to ISB, but with the downside of producing HDP incidences of similar frequency to ISB. Notably, anterior SSNB combined with an ICB produced significant reductions in PN palsy at lower volumes whilst still providing adequate analgesia, with studies suggesting that their minimum effective volumes are even lower. Further studies of this block combination are required to identify the minimum effective volumes in clinical practice and determine their true phrenic-sparing capabilities.

## Figures and Tables

**Table 1:** Summary of data from studies of the effect of modifications of the ISB on HDP incidence (*n* = 13).

Study	Approach	*n*	Local anaesthetic			Guidance	Total HDP incidence (%)
			Type	Volume (mL)	Concentration (%)		
Urmey and Gloeggler [72]	Lateral	10	Mepivacaine	20.0	1.50	Landmark (Winnie)	100.0
	Lateral	10	Mepivacaine	45.0	1.50	Landmark (Winnie)	100.0

Riazi et al. [73]	Lateral	20	Ropivacaine	5.0	0.50	US, NS	45.0
	Lateral	20	Ropivacaine	20.0	0.50	US, NS	100.0

Renes et al. [41]	Lateral	15	Ropivacaine	10.0	0.75	US	13.3
	Meier	15	Ropivacaine	10.0	0.75	NS	93.3

Lee et al. [74]	Lateral	30	Ropivacaine	5.0	0.75	US	33.0
	Lateral	30	Ropivacaine	10.0	0.75	US	60.0

Sinha et al. [75]	Lateral	15	Ropivacaine	10.0	0.50	US	93.3
	Lateral	15	Ropivacaine	20.0	0.50	US	93.3

Wong et al. [76]	Lateral	23	Ropivacaine	20.0	0.10	US, NS	34.8
	Lateral	24	Ropivacaine	20.0	0.20	US, NS	66.7

Jadon et al. [77]	Lateral	50	Ropivacaine	30.0	0.50	NS	16.0
	Lateral	50	Ropivacaine (+8 mg dexamethasone)	30.0	0.50	NS	20.0

Palhais et al. [30]	Lateral	20	Bupivacaine	20.0	0.50	US	90.0
	Extrafascial	19	Bupivacaine	20.0	0.50	US	21.0

Zhai et al. [78]	Lateral	30	Ropivacaine	10.0	0.50	US	66.7
	Lateral	30	Ropivacaine	20.0	0.25	US	70.0

Zhai et al. [79]	Lateral	33	Ropivacaine	6.7	0.75	US, NS	58.0
	Lateral	29	Ropivacaine	10.0	0.50	US, NS	69.0
	Lateral	33	Ropivacaine	20.0	0.25	US, NS	70.0

Meena et al. [80]	Lateral	35	Ropivacaine	10.0	0.50	NS	100.0
	Lateral	35	Ropivacaine	20.0	0.50	NS	100.0

Ayyanagouda [44]	Extrafascial	30	Ropivacaine	20.0	0.50	US, NS	17.0
	Lateral	30	Ropivacaine	20.0	0.50	US, NS	46.0

Kim et al. [35]	Lateral 2-point	26	Ropivacaine	10.0	0.50	US	53.8
	Lateral 2-point	26	Ropivacaine	15.0	0.50	US	92.3

US, ultrasound; NS, nerve stimulator.

**Table 2:** Summary of data from studies of the superior trunk block (*n* = 6).

Study	Needle position	*n*	Local anaesthetic			Median worst postoperative pain score	Total HDP incidence (%)
			Type	Volume (mL)	Concentration (%)		
Kang et al. [47]	Lateral	38	Ropivacaine (+5 *μ*g/ml epinephrine)	15	0.50	3	76.3

Kim et al. [49]	Posteroinferior (10 mL) and anterior (5 mL)	62	Bupivacaine	15	0.50	0	4.8

Kim et al. [48]	Lateral	35	Ropivacaine	5	0.50	5	14.3
	Lateral	35	Ropivacaine	15	0.50	3	65.7

Lee et al. [50]	Posterior (10 mL) and anterior (10 mL)	24	Lidocaine and ropivacaine	10, 10	2.00, 0.75	—	70.8

Robles et al. [81]	Posterior	30	Bupivacaine	12 ( ± up to 3 mL as required)	0.50	—	60.0

Zhang et al. [19]	Subparaneural	48	Ropivacaine	5	0.50	2	16.7

**Table 3:** Summary of data from studies of the suprascapular nerve block (*n* = 9).

Study	Approach	*n*	Combination	Local anaesthetic			HDP diagnostic method	Total HDP incidence (%)	Change in ipsilateral HD excursion (%)	FEV1 reduction (%)	FVC reduction (%)
				Type	Volume (mL)	Concentration (%)					
Trabelsi et al. [68]	Posterior	30	SCB (15 mL of bupivacaine 0.25%)	Bupivacaine	15	0.250	Symptoms	0.0			

Aliste et al. [67]	Posterior	20	ICB (10 mL levobupivacaine 0.25%), +epinephrine 5 g/mL	Levobupivacaine	10	0.250	US	0.0			

Auyong et al. [82]	Anterior	63	N	Ropivacaine	15	0.500	US, PFTs			13.0	10.0

Gianesello [83]	Anterior	19	ICB (levobupivacaine 0.5% 10 ml)	Levobupivacaine	10	0.500	PFTs			0.0	0.0

Taha et al. [84]	Anterior	36	N	Ropivacaine	25	0.500	US	5.6		0.0	

Ferré et al. [62]	Anterior	42	AXNB (10 mL 0.375% ropivacaine + 2 mg dexamethasone)	Ropivacaine	10	0.375	US	40.0	−17.20		
	Posterior	41	AXNB (10 mL 0.375% ropivacaine + 2 mg dexamethasone)	Ropivacaine	10	0.375	US	2.0	−3.60		

Lim et al. [85]	Anterior	20	N	Ropivacaine	15	0.500	US, PFTs		−1.80	7.0	3.6
	Posterior	20	N	Ropivacaine	15	0.500	US, PFTs		−1.20	5.3	6.8

Petroff et al. [86]	Anterior	24	N	Ropivacaine	10	1.000	US		−7.69		

Rhyner et al. [87]	Anterior	15	AXNB (10 ml bupivacaine 0.5%/adrenaline 1 : 200,000)	Bupivacaine	10	0.500	US, PFTs		−10.00	5.3	2.4

HD, hemidiaphragm; SCB, supraclavicular block; ICB, infraclavicular block.

## Data Availability

The data that support the findings of this study are available on request from the corresponding author.

## Supplementary Materials

Supplementary Materials Appendix A details the exact search strategies employed for each particular database search in order for our results to be replicated. Appendix B demonstrates the characteristics of every study included in our main text, including patient ASA status, procedures included in the study, and methods used to detect HDP.

## References

[1] Guo C. W., Ma J. X., Ma X. L. (2017). Supraclavicular block versus interscalene brachial plexus block for shoulder surgery: a meta-analysis of clinical control trials. *International Journal of Surgery*.

[2] Jain N. B., Higgins L. D., Losina E., Collins J., Blazar P. E., Katz J. N. (2014). Epidemiology of musculoskeletal upper extremity ambulatory surgery in the United States. *BMC Musculoskeletal Disorders*.

[3] Hughes M. S., Matava M. J., Wright R. W., Brophy R. H., Smith M. V. (2013). Interscalene brachial plexus block for arthroscopic shoulder surgery: a systematic review. *Journal of Bone and Joint Surgery American Volume*.

[4] Kumara A. B., Gogia A. R., Bajaj J. K., Agarwal N. (2016). Clinical evaluation of post-operative analgesia comparing suprascapular nerve block and interscalene brachial plexus block in patients undergoing shoulder arthroscopic surgery. *Journal of Clinical Orthopaedics and Trauma*.

[5] Fortier J., Chung F., Su J. (1998). Unanticipated admission after ambulatory surgery--a prospective study. *Canadian Journal of Anaesthesia*.

[6] Cubillos J., Giron-Arango L., Munoz-Leyva F. (2020). Diaphragm-sparing brachial plexus blocks: a focused review of current evidence and their role during the COVID-19 pandemic. *Current Opinion in Anaesthesiology*.

[7] Borgeat A. (2002). Anaesthesia for shoulder surgery. *Best Practice & Research Clinical Anaesthesiology*.

[8] Ghaleb A., D Dilley J. (2012). Anesthesia for shoulder surgery: a review of the interscalene block and a discussion of regional vs. general anesthesia. *The Open Anesthesiology Journal*.

[9] Miller E. M., Rider D., Waterman B. R. (2021). Editorial commentary: the evolution of regional anesthesia in arthroscopic rotator cuff repair: from throbbing shoulders to paralyzed diaphragms. *Arthroscopy: The Journal of Arthroscopic & Related Surgery*.

[10] Verelst P., van Zundert A. (2013). Respiratory impact of analgesic strategies for shoulder surgery. *Regional Anesthesia and Pain Medicine*.

[11] Tran D. Q., Layera S., Bravo D., Cristi-Sanchez I., Bermudez L., Aliste J. (2020). Diaphragm-sparing nerve blocks for shoulder surgery, revisited. *Regional Anesthesia and Pain Medicine*.

[12] Sun C., Ji X., Zhang X. (2021). Suprascapular nerve block is a clinically attractive alternative to interscalene nerve block during arthroscopic shoulder surgery: a meta-analysis of randomized controlled trials. *Journal of Orthopaedic Surgery and Research*.

[13] Nam Y. S., Panchal K., Kim I. B., Ji J. H., Park M. G., Park S. R. (2016). Anatomical study of the articular branch of the lateral pectoral nerve to the shoulder joint. *Knee Surgery, Sports Traumatology, Arthroscopy*.

[14] El-Boghdadly K., Chin K. J., Chan V. W. S. (2017). Phrenic nerve palsy and regional anesthesia for shoulder surgery. *Anesthesiology*.

[15] Aszmann O. C., Dellon A. L., Birely B. T., McFarland E. G. (1996). Innervation of the human shoulder joint and its implications for surgery. *Clinical Orthopaedics and Related Research*.

[16] Hewson D. W. (2019). Regional anaesthesia for shoulder surgery. *BJA Education*.

[17] Guanche C. A., Noble J., Solomonow M., Wink C. S. (1999). Periarticular neural elements in the shoulder joint. *Orthopedics*.

[18] Laumonerie P. (2020). Sensory innervation of the human shoulder joint: the three bridges to break. *Journal of Shoulder and Elbow Surgery*.

[19] Zhang H., Qu Z., Miao Y., Jia R., Li F., Hua Z. (2022). Comparison between subparaneural upper trunk and conventional interscalene blocks for arthroscopic shoulder surgery: a randomized noninferiority trial. *Anesthesia & Analgesia*.

[20] Jariwala A., Kumar B. C. R. P., Coventry D. M. (2014). Sudden severe postoperative dyspnea following shoulder surgery: remember inadvertent phrenic nerve block due to interscalene brachial plexus block. *International Journal of Shoulder Surgery*.

[21] Laumonerie P., Ferre F., Cances J. (2018). Ultrasound-guided proximal suprascapular nerve block: a cadaveric study. *Clinical Anatomy*.

[22] Kessler J., Schafhalter-Zoppoth I., Gray A. T. (2008). An ultrasound study of the phrenic nerve in the posterior cervical triangle: implications for the interscalene brachial plexus block. *Regional Anesthesia and Pain Medicine*.

[23] Moore K. L., Dalley A. F., Agur A. M. R. (2018). *Clinically Oriented Anatomy*.

[24] Marhofer P., Harrop-Griffiths W., Willschke H., Kirchmair L. (2010). Fifteen years of ultrasound guidance in regional anaesthesia: part 2-recent developments in block techniques. *British Journal of Anaesthesia*.

[25] Nason L. K., Walker C. M., McNeeley M. F., Burivong W., Fligner C. L., Godwin J. D. (2012). Imaging of the diaphragm: anatomy and function. *RadioGraphics*.

[26] Fell S. C. (1998). Surgical anatomy of the diaphragm and the phrenic nerve. *Chest Surgery Clinics of North America*.

[27] Bao X. X., Liu T., Feng H. R. (2021). The amplitude of diaphragm compound muscle action potential correlates with diaphragmatic excursion on ultrasound and pulmonary function after supraclavicular brachial plexus block. *Frontiers of Medicine*.

[28] Hartrick C. T., Tang Y. S., Siwek D., Murray R., Hunstad D., Smith G. (2012). The effect of initial local anesthetic dose with continuous interscalene analgesia on postoperative pain and diaphragmatic function in patients undergoing arthroscopic shoulder surgery: a double-blind, randomized controlled trial. *BMC Anesthesiology*.

[29] Urmey W. F., McDonald M. (1992). Hemidiaphragmatic paresis during interscalene brachial plexus block: effects on pulmonary function and chest wall mechanics. *Anesthesia & Analgesia*.

[30] Palhais N., Brull R., Kern C. (2016). Extrafascial injection for interscalene brachial plexus block reduces respiratory complications compared with a conventional intrafascial injection: a randomized, controlled, double-blind trial. *British Journal of Anaesthesia*.

[31] Kot Baixauli P., Rodriguez Gimillo P., Baldo Gosalvez J., de Andrés Ibáñez J. (2018). Usefulness of diaphragmatic ultrasound in the early diagnosis of phrenic nerve palsy after shoulder surgery in the prevention of post-operative respiratory complications. *Revista Espanola de Anestesiologia y Reanimacion*.

[32] Marty P., Ferre F., Basset B. (2018). Diaphragmatic paralysis in obese patients in arthroscopic shoulder surgery: consequences and causes. *Journal of Anesthesia*.

[33] Borgeat A., Levine M., Latmore M., Van Boxstael S., Blumenthal S. (2022). Interscalene brachial plexus block–landmarks and nerve stimulator technique. https://www.nysora.com/techniques/upper-extremity/intescalene/interscalene-brachial-plexus-block/.

[34] Winnie A. P. (1970). Interscalene brachial plexus block. *Anesthesia & Analgesia*.

[35] Kim K. S., Ahn J. H., Yoon J. H., Ji H. T., Kim I. S. (2021). Hemidiaphragmatic paresis following interscalene brachial plexus block with 2-point injection technique. *Pain Physician*.

[36] Raju P. K. B. C., Bowness J. S. (2022). Upper limb nerve blocks. *Anaesthesia and Intensive Care Medicine*.

[37] Gottardis M., Luger T., Florl C. (1993). Spirometry, blood gas analysis and ultrasonography of the diaphragm after Winnie’s interscalene brachial plexus block. *European Journal of Anaesthesiology*.

[38] Urmey W. F., Talts K. H., Sharrock N. E. (1991). One hundred percent incidence of hemidiaphragmatic paresis associated with interscalene brachial plexus anesthesia as diagnosed by ultrasonography. *Anesthesia & Analgesia*.

[39] Meier G., Bauereis C., Heinrich C. (1997). Der interskalenäre Plexuskatheter zur Anästhesie und postoperativen Schmerztherapie. *Anaesthesist, Der*.

[40] Sites B. D., Brull R. (2006). Ultrasound guidance in peripheral regional anesthesia: philosophy, evidence-based medicine, and techniques. *Current Opinion in Anaesthesiology*.

[41] Renes S. H., Rettig H. C., Gielen M. J., Wilder-Smith O. H., van Geffen G. J. (2009). Ultrasound-guided low-dose interscalene brachial plexus block reduces the incidence of hemidiaphragmatic paresis. *Regional Anesthesia and Pain Medicine*.

[42] Soeding P. F., Sha S., Royse C. F., Marks P., Hoy G., Royse A. G. (2005). A randomized trial of ultrasound-guided brachial plexus anaesthesia in upper limb surgery. *Anaesthesia & Intensive Care*.

[43] Albrecht E., Kirkham K. R., Taffé P. (2014). The maximum effective needle-to-nerve distance for ultrasound-guided interscalene block: an exploratory study. *Regional Anesthesia and Pain Medicine*.

[44] Ayyanagouda B. H. V. (2019). Hemi-diaphragmatic paresis following extrafascial versus conventional intrafascial approach for interscalene brachial plexus block: a double-blind randomised, controlled trial. *Indian Journal of Anaesthesia*.

[45] Burckett-St Laurent D., Chan V., Chin K. J. (2014). Refining the ultrasound-guided interscalene brachial plexus block: the superior trunk approach. *Canadian Journal of Anesthesia-Journal Canadien D Anesthesie*.

[46] Lin J. A., Chuang T. Y., Yao H. Y., Yang S. F., Tai Y. T. (2015). Ultrasound standard of peripheral nerve block for shoulder arthroscopy: a single-penetration double-injection approach targeting the superior trunk and supraclavicular nerve in the lateral decubitus position. *British Journal of Anaesthesia*.

[47] Kang R., Jeong J. S., Chin K. J. (2019). Superior trunk block provides noninferior analgesia compared with interscalene brachial plexus block in arthroscopic shoulder surgery. *Anesthesiology*.

[48] Kim H., Han J. U., Lee W. (2021). Effects of local anesthetic volume (standard versus low) on incidence of hemidiaphragmatic paralysis and analgesic quality for ultrasound-guided superior trunk block after arthroscopic shoulder surgery. *Anesthesia & Analgesia*.

[49] Kim D. H., Lin Y., Beathe J. C. (2019). Superior trunk block: a phrenic-sparing alternative to the interscalene block: a randomized controlled trial. *Anesthesiology*.

[50] Lee M. G., Shin Y. J., You H. S., Lim C. H., Chang Y. J., Shin H. J. (2021). A comparison of anesthetic quality between interscalene block and superior trunk block for arthroscopic shoulder surgery: a randomized controlled trial. *Pain Physician*.

[51] Sakamoto Y. (2012). Spatial relationships between the morphologies and innervations of the scalene and anterior vertebral muscles. *Annals of Anatomy-Anatomischer Anzeiger*.

[52] Cros Campoy J., Domingo Bosch O., Pomes J., Lee J., Fox B., Sala-Blanch X. (2019). Upper trunk block for shoulder analgesia with potential phrenic nerve sparing: a preliminary anatomical report. *Regional Anesthesia and Pain Medicine*.

[53] Wertheim H., Rovenstine E. A. (1941). Suprascapular nerve block. *Anesthesiology*.

[54] Risdall J. E., Sharwood-Smith G. H. (1992). Suprascapular nerve block. New indications and a safer technique. *Anaesthesia*.

[55] Warner J. P., Krushell R. J., Masquelet A., Gerber C. (1992). Anatomy and relationships of the suprascapular nerve: anatomical constraints to mobilization of the supraspinatus and infraspinatus muscles in the management of massive rotator-cuff tears. *The Journal of Bone and Joint Surgery*.

[56] Faruch Bilfeld M., Lapègue F., Sans N., Chiavassa Gandois H., Laumonerie P., Larbi A. (2017). Ultrasonography study of the suprascapular nerve. *Diagnostic and Interventional Imaging*.

[57] Maikong N., Mahakkanukrauh P. (2021). Review of suprascapular nerve block under ultrasound-guided: alternative technique with clinical roles. *International Medical Journal*.

[58] Siegenthaler A., Moriggl B., Mlekusch S. (2012). Ultrasound-guided suprascapular nerve block, description of a novel supraclavicular approach. *Regional Anesthesia and Pain Medicine*.

[59] Sehmbi H., Johnson M., Dhir S. (2019). Ultrasound-guided subomohyoid suprascapular nerve block and phrenic nerve involvement: a cadaveric dye study. *Regional Anesthesia and Pain Medicine*.

[60] Ritchie E. D., Tong D., Chung F., Norris A. M., Miniaci A., Vairavanathan S. D. (1997). Suprascapular nerve block for postoperative pain relief in arthroscopic shoulder surgery: a new modality?. *Anesthesia & Analgesia*.

[61] Harmon D., Hearty C. (2007). Ultrasound-guided suprascapular nerve block technique. *Pain Physician*.

[62] Ferré F., Pommier M., Laumonerie P. (2020). Hemidiaphragmatic paralysis following ultrasound-guided anterior vs. posterior suprascapular nerve block: a double-blind, randomised control trial. *Anaesthesia*.

[63] Blasco L., Laumonerie P., Tibbo M. (2020). Ultrasound-guided proximal and distal suprascapular nerve blocks: a comparative cadaveric study. *Pain Medicine*.

[64] Chan C. W., Peng P. W. (2011). Suprascapular nerve block: a narrative review. *Regional Anesthesia and Pain Medicine*.

[65] Price D. J. (2007). The shoulder block: a new alternative to interscalene brachial plexus blockade for the control of postoperative shoulder pain. *Anaesthesia & Intensive Care*.

[66] Gonzalez-Arnay E., Jimenez-Sanchez L., Garcia-Simon D. (2020). Ultrasonography-guided anterior approach for axillary nerve blockade: an anatomical study. *Clinical Anatomy*.

[67] Aliste J., Bravo D., Finlayson R. J., Tran D. Q. (2018). A randomized comparison between interscalene and combined infraclavicular-suprascapular blocks for arthroscopic shoulder surgery. *Canadian Journal of Anesthesia/Journal canadien d’anesthésie*.

[68] Trabelsi W., Ben Gabsia A., Lebbi A., Sammoud W., Labbene I., Ferjani M. (2017). Suprascapular block associated with supraclavicular block: an alternative to isolated interscalene block for analgesia in shoulder instability surgery?. *Orthopaedics & Traumatology-Surgery & Research*.

[69] Kaye A. D., Allampalli V., Fisher P. (2021). Supraclavicular vs. Infraclavicular brachial plexus nerve blocks: clinical, pharmacological, and anatomical considerations. *Anesthesiology and Pain Medicine*.

[70] Tran D. Q. H., Elgueta M. F., Aliste J., Finlayson R. J. (2017). Diaphragm-sparing nerve blocks for shoulder surgery. *Regional Anesthesia and Pain Medicine*.

[71] Page M. J., McKenzie J. E., Bossuyt P. M. (2021). The PRISMA 2020 statement: an updated guideline for reporting systematic reviews. *BMJ*.

[72] Urmey W. F., Gloeggler P. J. (1993). Pulmonary function changes during interscalene brachial plexus block: effects of decreasing local anesthetic injection volume. *Regional Anesthesia*.

[73] Riazi S., Carmichael N., Awad I., Holtby R. M., McCartney C. J. L. (2008). Effect of local anaesthetic volume (20 vs 5 ml) on the efficacy and respiratory consequences of ultrasound-guided interscalene brachial plexus block. *British Journal of Anaesthesia*.

[74] Lee J. H., Cho S. H., Kim S. H. (2011). Ropivacaine for ultrasound-guided interscalene block: 5 mL provides similar analgesia but less phrenic nerve paralysis than 10 mL. *Canadian Journal of Anesthesia-Journal Canadien D Anesthesie*.

[75] Sinha S. K., Abrams J. H., Barnett J. T. (2011). Decreasing the local anesthetic volume from 20 to 10 mL for ultrasound-guided interscalene block at the cricoid level does not reduce the incidence of hemidiaphragmatic paresis. *Regional Anesthesia and Pain Medicine*.

[76] Wong A. K., Keeney L., Chen L., Williams R., Liu J., Elkassabany N. M. (2016). Effect of local anesthetic concentration (0.2% vs 0.1% ropivacaine) on pulmonary function, and analgesia after ultrasound-guided interscalene brachial plexus block: a randomized controlled study. *Pain Medicine*.

[77] Jadon A., Dixit S., Kedia S. K., Chakraborty S., Agrawal A., Sinha N. (2015). Interscalene brachial plexus block for shoulder arthroscopic surgery: prospective randomised controlled study of effects of 0.5% ropivacaine and 0.5% ropivacaine with dexamethasone. *Indian Journal of Anaesthesia*.

[78] Zhai W., Wang X., Li M., Guo X. (2016a). A study on diaphragm function after interscalene brachial plexus block using a fixed dose of ropivacaine with different concentrations. *Zhonghua Yixue Zazhi*.

[79] Zhai W. W., Wang X. D., Rong Y. L., Li M., Wang H. (2016b). Effects of a fixed low-dose ropivacaine with different volume and concentrations on interscalene brachial plexus block: a randomized controlled trial. *BMC Anesthesiology*.

[80] Meena D., Sahu G., Saini S., Aravindan A., Datta P. (2018). Comparison of two different volumes of ropivacaine used in nerve stimulator guided inter-scalene block for arthroscopic shoulder surgery; A randomized controlled trial. *Anesthesia: Essays and Researches*.

[81] Robles C., Berardone N., Orebaugh S. (2022). Effect of superior trunk block on diaphragm function and respiratory parameters after shoulder surgery. *Regional Anesthesia and Pain Medicine*.

[82] Auyong D. B., Hanson N. A., Joseph R. S., Schmidt B. E., Slee A. E., Yuan S. C. (2018). Comparison of anterior suprascapular, supraclavicular, and interscalene nerve block approaches for major outpatient arthroscopic shoulder surgery. *Anesthesiology*.

[83] Gianesello L. (2018). Respiratory effect of interscalene brachial plexus block vs combined infraclavicular plexus block with suprascapular nerve block for arthroscopic shoulder surgery. *Journal of Clinical Anesthesia*.

[84] Taha A. M., Yurdi N. A., Elahl M. I., Abd-Elmaksoud A. M. (2019). Diaphragm-sparing effect of the infraclavicular subomohyoid block vs low volume interscalene block. A randomized blinded study. *Acta Anaesthesiologica Scandinavica*.

[85] Lim Y. C., Koo Z. K., Ho V. W., Chang S. S., Manohara S., Tong Q. J. (2020). Randomized, controlled trial comparing respiratory and analgesic effects of interscalene, anterior suprascapular, and posterior suprascapular nerve blocks for arthroscopic shoulder surgery. *Korean Journal of Anesthesiology*.

[86] Petroff D., Wiegel M., Pech V., Salz P., Mrongowius J., Reske A. W. (2020). Differential lung ventilation assessed by electrical impedance tomography in ultrasound-guided anterior suprascapular nerve block vs. interscalene brachial plexus block: a patient and assessor-blind, randomised controlled trial. *European Journal of Anaesthesiology*.

[87] Rhyner P., Kirkham K., Hirotsu C., Farron A., Albrecht E. (2020). A randomised controlled trial of shoulder block vs. interscalene brachial plexus block for ventilatory function after shoulder arthroscopy. *Anaesthesia*.

[88] Fredrickson M. J., Smith K. R., Wong A. C. (2010). Importance of volume and concentration for ropivacaine interscalene block in preventing recovery room pain and minimizing motor block after shoulder surgery. *Anesthesiology*.

[89] Kawai H. A. K. H. (2000). *Brachial Plexus Palsy*.

[90] Cornish P. B., Leaper C. (2006). The sheath of the brachial plexus: fact or fiction?. *Anesthesiology*.

[91] Orebaugh S. L., Williams B. A. (2009). Brachial plexus anatomy: normal and variant. *TheScientificWorldJOURNAL*.

[92] Yang W. T., Chui P. T., Metreweli C. (1998). Anatomy of the normal brachial plexus revealed by sonography and the role of sonographic guidance in anesthesia of the brachial plexus. *American Journal of Roentgenology*.

[93] Thompson G. E., Rorie D. K. (1983). Functional anatomy of the brachial plexus sheaths. *Anesthesiology*.

[94] Taha A. M. (2017). ISO (Infraclavicular-SubOmohyoid) block: a single-puncture technique for diaphragm- and opioid-sparing shoulder anaesthesia. *British Journal of Anaesthesia*.

[95] Petrar S. D., Seltenrich M. E., Head S. J., Schwarz S. K. (2015). Hemidiaphragmatic paralysis following ultrasound-guided supraclavicular versus infraclavicular brachial plexus blockade: a randomized clinical trial. *Regional Anesthesia and Pain Medicine*.

[96] Hussain N., Goldar G., Ragina N., Banfield L., Laffey J. G., Abdallah F. W. (2017). Suprascapular and interscalene nerve block for shoulder surgery: a systematic review and meta-analysis. *Anesthesiology*.

[97] White L., Reardon D., Davis K., Velli G., Bright M. (2022). Anterior suprascapular nerve block versus interscalene brachial plexus block for arthroscopic shoulder surgery: a systematic review and meta-analysis of randomized controlled trials. *Journal of Anesthesia*.

[98] Maikong N., Kantakam P., Sinthubua A., Mahakkanukrauh P., Tran D. Q., Leurcharusmee P. (2021). Cadaveric study investigating the phrenic-sparing volume for anterior suprascapular nerve block. *Regional Anesthesia and Pain Medicine*.

