# Supplementary material for: Impact of Peripheral Nerve Block Technique on Incidence of Phrenic Nerve Palsy in Shoulder Surgery
Source: Anesthesiol Res Pract. 2023 Sep 11;2023:9962595. doi: 10.1155/2023/9962595 (PMC10506885; doi:10.1155/2023/9962595)
Supplement: Supplementary Materials — Appendix A details the exact search strategies employed for each particular database search in order for our results to be replicated. Appendix B demonstrates the characteristics of every study included in our main text, including patient ASA status, procedures included in the study, and methods used to detect HDP. [file 9962595.f1.docx]

**SUPPLEMENTARY MATERIALS**

**Appendix A – Search Strategies**

**Search Strategy for OVID Medline**

1. nerve block/
2. shoulder/
3. interscalene.mp.
4. isb.mp.
5. suprascapular.mp.
6. ssnb.mp.
7. superior trunk.mp.
8. shoulder block.mp.
9. upper trunk.mp.
10. brachial plexus/
11. 1 and 2
12. 3 or 4 or 5 or 6 or 7 or 8 or 9 or 10
13. 11 and 12
14. phrenic nerve/
15. diaphragm/
16. hemidiaphragm/ or diaphragm paralysis/
17. pulmonary.mp.
18. ventilation.mp.
19. respiratory.mp.
20. 14 or 15 or 16 or 17 or 18 or 19
21. 13 and 20

**Search Strategy for OVID Embase**

1. nerve block/
2. shoulder/
3. interscalene.mp.
4. isb.mp.
5. suprascapular.mp.
6. ssnb.mp.
7. superior trunk.mp.
8. shoulder block.mp.
9. upper trunk.mp.
10. brachial plexus/
11. 1 and 2
12. 3 or 4 or 5 or 6 or 7 or 8 or 9 or 10
13. 11 and 12
14. phrenic nerve/
15. diaphragm/
16. hemidiaphragm/ or diaphragm paralysis/
17. pulmonary.mp.
18. ventilation.mp.
19. respiratory.mp.
20. 14 or 15 or 16 or 17 or 18 or 19
21. 13 and 20

**Search Strategy for Web of Science**

**nerve block** (All Fields) and **shoulder** (All Fields) and **interscalene or isb or ssnb or suprascapular or shoulder block or "superior trunk" or "upper trunk" or "brachial plexus"** (All Fields) and **diaphragm* or phrenic or hemidiaphragm* or pulmonary or ventilation or respiratory** (All Fields)

**Search Strategy for Scopus**

(TITLE-ABS-KEY (“nerve block" )  AND  TITLE-ABS-KEY ( shoulder )  AND  TITLE-ABS-KEY ( interscalene  OR  isb  OR  ssnb  OR  suprascapular  OR  "shoulder block"  OR  "superior trunk"  OR  "upper trunk"  OR  "brachial plexus" )  AND  TITLE-ABS-KEY ( phrenic  OR  diaphragm  OR  hemidiaphragm*  OR  respiratory  OR  ventilation  OR  pulmonary ) )

**Search Strategy for Cochrane Library**

1. nerve block/
2. shoulder/
3. interscalene.mp.
4. isb.mp.
5. suprascapular.mp.
6. ssnb.mp.
7. superior trunk.mp.
8. shoulder block.mp.
9. upper trunk.mp.
10. brachial plexus/
11. 1 and 2
12. 3 or 4 or 5 or 6 or 7 or 8 or 9 or 10
13. 11 and 12
14. phrenic nerve/
15. diaphragm/
16. hemidiaphragm/ or diaphragm paralysis/
17. pulmonary.mp.
18. ventilation.mp.
19. respiratory.mp.
20. 14 or 15 or 16 or 17 or 18 or 19
21. 13 and 20

**Appendix B – Study Characteristics**

**TABLE I.** **Summary of interscalene block study characteristics (n = 13).**

| **Study** | **ASA Status** | **General Anaesthetic** | **Procedure** | **HDP Diagnostic Method** |
| --- | --- | --- | --- | --- |
| **Urmey and Gloeggler (1993)** | - | N | "Elective Shoulder Surgery" | US, PFTs |
| **Riazi et al. (2008)** | I-III | Y | "Shoulder Surgery" | US, PFTs |
| **Renes et al. (2009)** | I-III | Y | "Elective Shoulder Surgery" | US, PFTs |
| **Lee et al. (2011)** | - | Y | Arthroscopy, Rotator Cuff Repair | XR |
| **Sinha et al. (2011)** | - | Y | Arthroscopy | US, PFTs |
| **Elkassabany et al. (2015)** | I-III | Y | Arthroscopy, Rotator Cuff Repair | US, PFTs |
| **Jadon et al. (2015)** | I-II | N | Arthroscopy | Symptoms |
| **Palhais et al. (2016)** | I-III | Y | “Shoulder and Clavicle Surgery” | US, PFTs |
| **Zhai et al. (2016a)** | - | Y | Arthroscopy | US, PFTs |
| **Zhai et al. (2016b)** | I-II | Y | Arthroscopy | US |
| **Sahu et al. (2018)** | I-II | Y | Arthroscopy | XR |
| **Ayyanagouda (2019)** | I-III | N | “Proximal upper limb surgery” | US, PFTs |
| **Kim et al. (2021b)** | Not given | Y | Brisement Manipulation or Arthroscopy | US |

Abbreviations: ASA, American Society of Anesthesiologists; US, ultrasound; PFTs, pulmonary function tests; XR, x-ray

**TABLE II. Summary of superior trunk block study characteristics (n = 6)**

| **Study** | **ASA Status** | **General Anaesthetic** | **Procedure** | **HDP Diagnostic Method** |
| --- | --- | --- | --- | --- |
| **Kang et al. (2019)** | I-III | Y | Arthroscopy | US, PFTs |
| **Kim et al. (2019)** | I-III | Y | Arthroscopy | US |
| **Kim et al. (2021a)** | I-III | Y | Arthroscopy | US, PFTs |
| **Lee et al. (2021)** | I-II | N | Arthroscopy (Rotator Cuff Syndrome) | US |
| **Robles et al. (2022)** | I-III | N | Arthroscopy | US |
| **Zhang et al. (2022)** | I-II | Y | Arthroscopy | US |

**TABLE III. Summary of suprascapular nerve block study characteristics (n = 9)**

| **Study** | **ASA Status** | **General Anaesthetic** | **Procedure** | **HDP Diagnostic Method** |
| --- | --- | --- | --- | --- |
| **Trabelsi et al. (2017)** | I-II | Y | Shoulder Instability Surgery | Symptoms |
| **Aliste et al. (2018)** | I-III | Y | Arthroscopy | US |
| **Renes et al. (2009)** | I-III | Y | Arthroscopy | US, PFTs |
| **Gianesello (2018)** | I-II | N | Arthroscopy | PFTs |
| **Taha et al. (2019)** | I-III | Y | Arthroscopy | US |
| **Ferré et al. (2020)** | I-III | Y | Arthroscopy | US |
| **Lim et al. (2020)** | I-III | Y | Arthroscopy | US, PFTs |
| **Petroff et al. (2020)** | I-III | Y | Arthroscopy | US |
| **Rhyner et al. (2020)** | I-III | Y | Arthroscopy | US, PFTs |
